# Comparison of Endoscopic Submuscosal Implantation vs. Surgical Intramuscular Implantation of VX2 Fragments for Establishing a Rabbit Esophageal Tumor Model for Mimicking Human Esophageal Squamous Carcinoma

**DOI:** 10.1371/journal.pone.0085326

**Published:** 2014-01-24

**Authors:** Jin Huang, Yin Zhang, Hengao Zhong, Zhining Fan, Guobin Jiang, Yingzhou Shen, Hanming Song, Zhijian Tao, Kuangjing Wang

**Affiliations:** 1 Digestive Medical Center, The Second Affiliated Hospital of Nanjing Medical University, Nanjing, Jiangsu Province, China; 2 Division of Digestive Diseases, Renming Hospital of Ma Anshan, Ma Anshan City, Anhui Province, China; Peter MacCallum Cancer Centre, Australia

## Abstract

**Purpose:**

This study was undertaken to establish a rabbit esophageal tumor model for mimicking human esophageal squamous carcinoma (ESC) by endoscopic and surgical implantation of VX2 tumors.

**Methods:**

Fragments of a VX2 tumour were endoscopically implanted in the submucosal layer of the thoracic esophagus of 32 New Zealand white rabbits, while 34 animals received surgical implantation into the muscular layer. Then, the animals were studied endoscopically and pathologically. The safety and efficiency of the two methods and the pathological features of the animal models were analyzed.

**Results:**

Both the endoscopic and the surgical method had a relatively high success rate of tumor implantation [93.7% (30/32) vs. 97.1% (33/34)] and tumor growth [86.7% (26/30) vs. 81.8% (27/33)], and the variation in the results was not statistically significant (*P*>0.05). Compared with those produced by the surgical method, the models produced by the endoscopic method had a higher rate of severe esophageal stricture [61.5% (16/26) vs. 29.6% (8/27)] and of intra-luminal tumor growth [73.1% (19/26) vs. 37.0% (10/27)], and had a lower rate of tumor invasion of adjacent organs [53.8% (14/26) vs. 81.5% (22/27)]; all of these results were statistically significant (P<0.05). However, the difference in the survival time and the rates of tumor regional/distant metastasis [38.5% (10/26) vs. 51.8% (14/27)] between the two methods were not statistically significant (P>0.05).

**Conclusion:**

The endoscopic and surgical methods are both safe and effective for establishment of VX2 tumors in the rabbit esophagus. The models produced by the two methods have different pathologic features mimicking that of human ESC. We recommend the models for studies on surgical procedures and minimally invasive treatments.

## Introduction

The annual incidence of esophageal cancer is as high as 30 to 800 per 100,000 people. Although the most common esophageal cancer in developed countries is adenocarcinoma, esophageal squamous carcinoma (ESC) remains an important clinical problem in China, Iran and parts of Africa [1], [2]. For patients with advanced ESC, new therapeutic strategies are needed to prolong the survival time and improve the quality of life. However, the progress in relevant research is limited by the lack of a suitable moderate-to-large-sized animal model [3].

Although small animal model for esophageal cancer, such as rodent models, are available and wildly used in research [4]–[6], moderate-to-large-sized animal models are still desperately needed for preclinical studies. Theoretically, all therapeutic strategies that are systematically attempted in humans should be based on prior knowledge that has been conceived by sound science and tested in a model that has a reasonable chance of predicting at least the safety of the maneuver. Compared with the rodent tumor model, moderate-to-large-sized animal models can better simulate the human body environment and allow for research on surgical operations and minimally invasive treatments [7]–[9] such as endoscopic therapy, stent placement and intravascular interventional treatments.

The VX2 tumor model, in which the carcinoma is developed from papillomas in domestic rabbits and is proven histologically to be the squamous cell type, was originally proposed by Shope and Hurst in 1933 [10], [11], and is now used for studies involving many important organs in the human body, such as the liver, the lung, the kidney, the head and neck [12]–[14]. Rabbits are a moderate-to-large-sized animal, but they have rarely been used as an esophageal VX2 tumor model. Therefore, an effective modeling method and the pathological characteristics of the relevant model have not been introduced.

In this study, we endoscopically implanted VX2 fragments to the submuscosal layer and surgically into the muscular layer of thoracic esophagus of rabbits, and successfully established esophageal tumor models with the two methods. We investigated the safety and efficiency of the two methods, as well as the pathological characteristics of the models to provide detailed information on models and modeling methods for future research.

## Materials and Methods

### Animals and Tumors

A total of 66 male and female New Zealand White rabbits weighing between 2.5 and 3.0 kg were used in this study. Animals were obtained from the Jiangsu Agricultural Academy of Science in China and were allowed free access to food and water. The VX2 tumor was obtained from the Surgery Department in the First Affiliated Hospital of Nanjing Medical University.

This study was carried out in strict accordance with the recommendations in the Guide for the Care and Use of Laboratory Animals of the National Institutes of Health. The protocol was approved by the Animal Care and Use Subcommittee at Nanjing Medical University. All surgeries were performed under sodium pentobarbital anesthesia, and all efforts were made to minimize suffering.

### Tumor propagation and maintenance

Rabbits with hind limb tumors (donors) were used to propagate and maintain VX2 tumors. Approximately two to three of thawed VX2 tumor tissue fragments (approximately 0.5 mm^3^), previously stored in liquid nitrogen, were resuscitated and injected deep into the hind limb gluteal muscles of rabbits anesthetized by intramuscular injection of a 35-mg/kg pentobarbital sodium (Sigma Chemical Co., St. Louis, MO, USA). Three weeks after implantation, animals were sacrificed and hind limb tumors were harvested and processed immediately. The VX2 tumors were cleaned from the surrounding tissue and the gross necrotic portions of tumors were removed.

The collected tumors were cut into small pieces (approximately 0.5 mm^3^) and preserved in saline for implantation: approximately 0.3 ml of saline solution with four pieces of tumor fragments were placed in a 2-ml syringe on ice until they were injected into the recipient rabbit's esophagus.

### Endoscopic implantation of VX2 fragments

**Figure 1:**
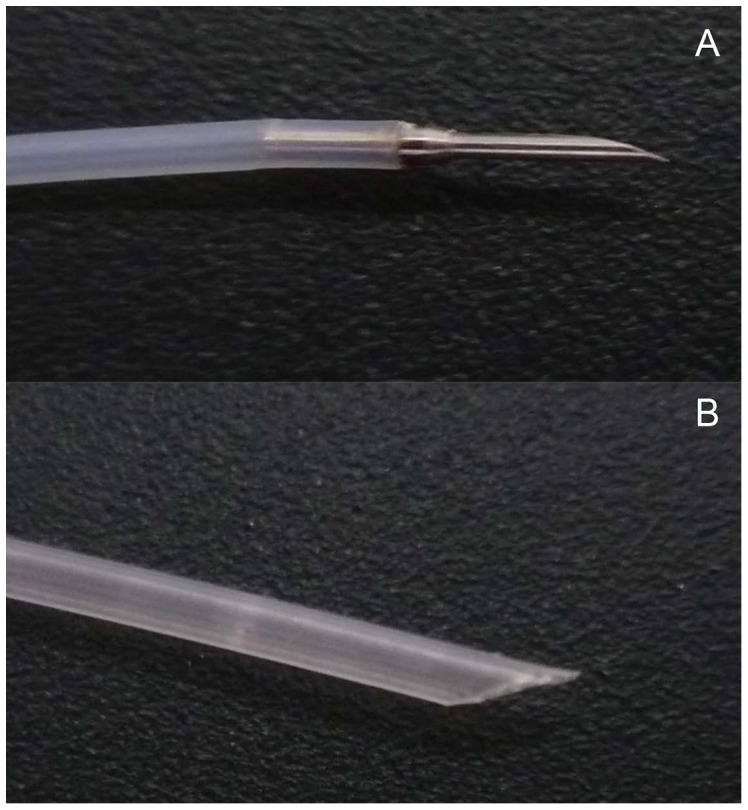
Needles for endoscopic implantation. (A) Tip of the endoscopic needle (fine needle). (B) Tip of the revised needle (large needle).

After 24 h of fasting with free access to water, the rabbits were anesthetized (as noted above) and then were placed in the left lateral decubitus position. The implantation site was approximately 5 cm away from the cardia, where was the equivalent of 15 cm away from the incisors of the rabbit. A slim endoscope (Olympus GIFXP260, Japan) was inserted into the thoracic esophagus. Using a fine needle (the inner core of an endoscopic needle, Olympus, MAJ-68, 23-gauge, Fig. 1A), 0.5 ml of saline was injected into the esophageal submucosal layer to elevate the mucosa layer. After that, a puncture was made across the mucosa into the submucosal layer using a large needle (a revised endoscopic needle, Olympus, MAJ-68, with an oblique cut in the front, Fig. 1B) and the success of the puncture was confirmed by further elevation of the mucosa when saline was injected. Approximately 0.3 ml of saline containing four VX2 pieces was then injected into the submucosal layer of the rabbits (Video S1).

### Surgical implantation of VX2 tumors

**Figure 2:**
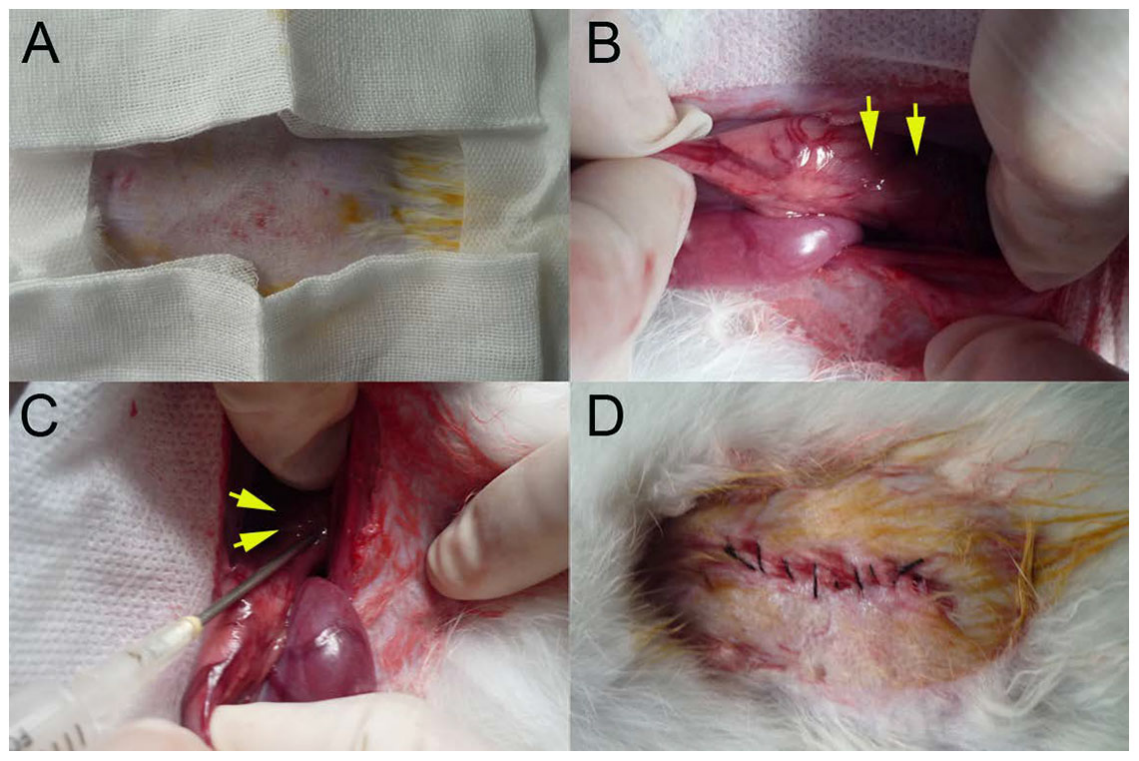
Procedure for surgical implantation. (A) Hair removal for the operation. (B)The lower thoracic esophagus (yellow arrows) was pulled into the abdominal cavity. (C) Using a 20 G needle, VX2 fragments were inoculated (yellow arrows) into the muscular layer of the esophagus. (D) The abdominal wall was closed.

The rabbits were anesthetized (as mentioned previously) in the supine position and the hair over the abdominal region was molted with 8% sodium sulfide. Then, the abdominal region was cleaned with saline, which was followed by the disinfection of the operating site. The abdominal cavity was opened by the subxiphoid process, and the lower thoracic esophagus was pulled into the abdominal cavity and exposed. The implantation site was approximately 4–5 cm away from the cardia of the rabbit. A needle (20 G) was inserted into the muscular layer of the esophagus and VX2 fragments were injected (Fig. 2). As soon as the needle withdrew, manual pressure was applied to the surface of the esophageal wound for approximately 3 minutes to avoid the fragments falling out of the incision. Next the abdominal wall was closed in two layers. After the operation, the wound was kept sterile, and the antibiotic gentamicin (2.5mg/kg) was injected into the muscles to prevent infection of the wound and ensure wound healing.

### Endoscopic follow up

All animals in this study received the implantation procedure only once, regardless of the implantation method. After implantation, an endoscopic examination was performed once a week to observe the tumor growth and the degree of esophageal stenosis. The anesthesia procedure was the same as that described before.

A diagnostic criteria for tumor growth was used in this study: tumor growth was determined by an endoscopic biopsy or by histologic sections. We conducted a biopsy when the diameter of the tumor at the widest part was larger than or equal to 0.3 cm under endoscopy. If the tumor diameter was less than 0.3 cm under endoscopy, we would depend on autopsy at the endpoint to prove tumor growth. If no sign of tumor intra-luminal growth or extrinsic compression occurred over four weeks, the rabbit was regarded as a failure animal and was dropped out of the study.

Three grades were used to evaluate the esophageal stenosis under endoscope: mild stenosis for tumors less than or equal to 1/3 of the luminal diameter; moderate stenosis for tumors more than 1/3 but less than or equal to 2/3 of the luminal diameter and severe stenosis for tumors more than 2/3 of the luminal diameter.

### Survival time and Necropsy

Survival time was recorded for all of the rabbits with tumor growth, and the diagnostic criteria for tumor growth was as already described.

A humane endpoint was used in this study, at which the animal models would be humanely sacrificed when they would not eat any food or water for several days and their health was so weak that they could not stand.

Rabbits were fed separately in cages to observe daily the quantity of ingested food and water. Paste food or liquid food (vegetable juice) was provided for animals with dysphagia or a 50% reduction in food intake. The euthanasia procedure was as follows: the animal was first anesthetized by an intramuscular injection of a 35-mg/kg pentobarbital sodium through the ear venous and then was injected with 20 ml of air.

The tumor growth patterns including intra-luminal and extra-luminal growth were detected by endoscopy and necropsy. Tumor intra-luminal growth was defined as the condition when the size of the tumor body in the esophageal lumen was larger than its size outside of the esophageal lumen. Tumor extra-luminal growth was defined as the conditions when the size of the tumor body in the esophageal lumen was less than its size outside of the esophageal lumen.

Gross evidence for tumors was detected in organs such as the lung, pleura, pericardium, heart, trachea/bronchus, diaphragm, peritoneum, liver and kidney, and sections of the tissues associated with tumor invasion or metastasis were examined using hematoxylin and eosin (H&E) staining.

### Data analysis

We calculated success rate of the implantation and the tumor growth, and the percentage of the varying degrees of esophageal stenosis and the patterns of tumor growth. The rates of tumor invasion into adjacent organs and regional/distant metastasis were also analyzed. The chi square test was used to determine the statistical significance of the differences between groups. The Kaplan-Meier method with log rank test was used to compare the survival. The statistical significance was set at the *P*<0.05 level. All statistical tests were performed with the software SPSS 16.0.

## Results

### Success rate of tumor implantation and tumor growth

Among the 66 animals that received VX2 fragments implantation, 32 rabbits received endoscopic implantation and 34 received surgical implantation. Two rabbits (6.3%) died of esophageal perforations from the endoscopic procedure, and one rabbit (2.9%) died from severe injury during surgical implantation. The success rates of tumor implantation for the two groups were 93.7% (30/32) and 97.1% (33/34), respectively. Four weeks after implantation, tumor growth was observed in 26/30 (86.7%) rabbits that received endoscopic implantation and in 27/33 (81.8%) rabbits that received surgical implantation. The difference in the success rates of implantation and tumor growth between the two methods was not statistically significant (*P*>0.05).

### Degree of esophageal stricture

**Figure 3:**
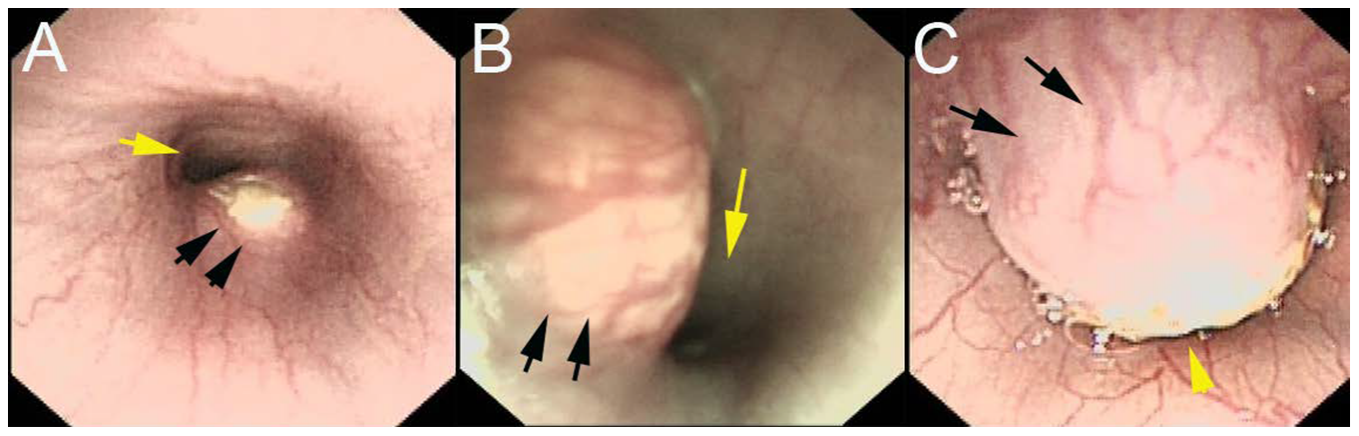
The degree of esophageal stenosis. Endoscopic view of (A) mild stricture, (B) moderate stricture and (C) severe stricture in a rabbit model produced by the endoscopic method. The black arrows point to the tumor and the yellow arrow points to the esophageal lumen.

During the lifetimes of the animals, severe stricture was observed in 16/26 (61.5%) rabbits that received endoscopic implantation and in 8/27 (29.6%) rabbits that received surgical inoculation; mild-to-moderate stricture was observed in 10/26 (38.5%) and 19/27 (70.4%) rabbits in the two groups, respectively (Fig. 3). The difference in the rates of severe esophageal stricture between the two groups was 31.9% (95% CI, 6.5% to 57.3%). The results were statistically significant (*P* = 0.019).

### Tumor growth patterns

**Figure 4:**
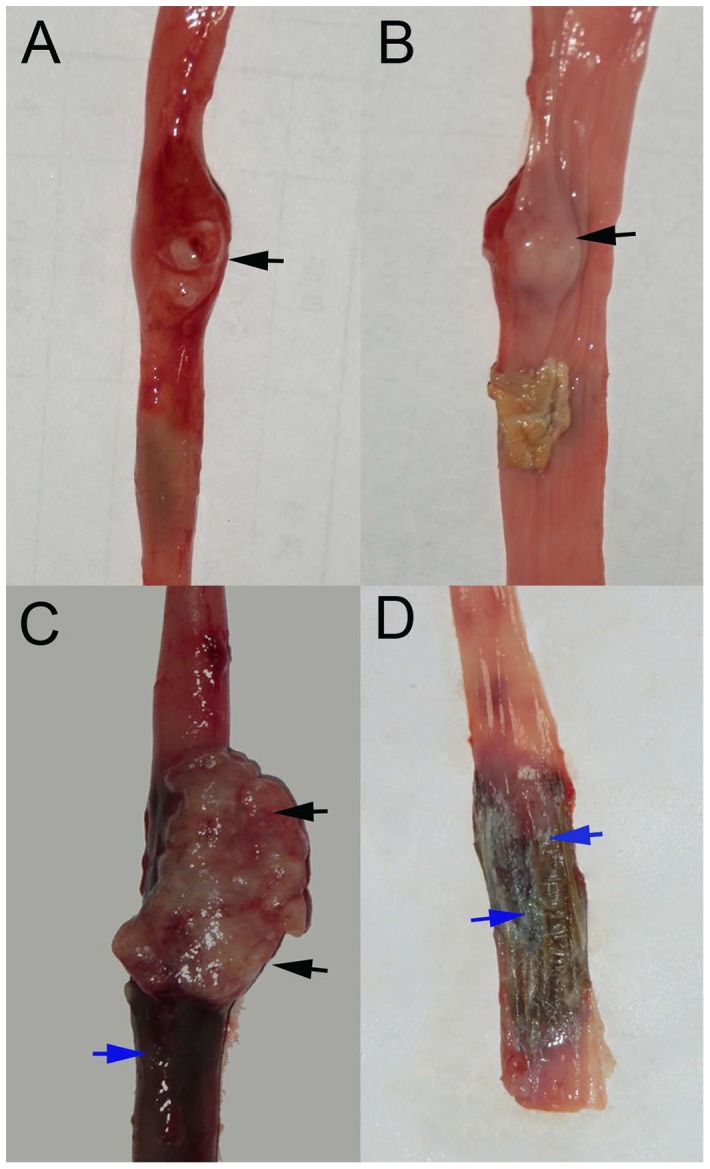
The tumor growth patterns. Tumor intra-luminal growth: (A) macroscopic external view of the esophageal tumor (black arrow) and (B) internal view of the esophageal tumor (black arrow) in a sacrificed animal model produced by the endoscopic method. Tumor extra-luminal growth: (C) macroscopic view of the tumor (black arrows) with esophageal congestion (blue arrow) from the external esophagus in a sacrificed animal model produced by the surgical method, and (D) macroscopic view of no tumor but esophageal congestion (blue arrows) from the internal esophagus.

Tumor intra-luminal growth (Fig. 4A, B) was detected in 19/26 (73.1%) rabbits that received endoscopic implantation and in 10/27 (37.0%) rabbits that received surgical implantation, whereas 7/26 (26.9%) and 17/27 (63.0%) rabbits had extra-luminal tumors (Fig. 4C, D) in the two groups, respectively. The difference in the rates of intra-luminal growth between the two groups was 36.1% (95% CI, 11.2% to 59.8%) and the results were statistically significant (*P* = 0.008).

### Tumor invasion of adjacent tissue and metastasis

**Figure 5:**
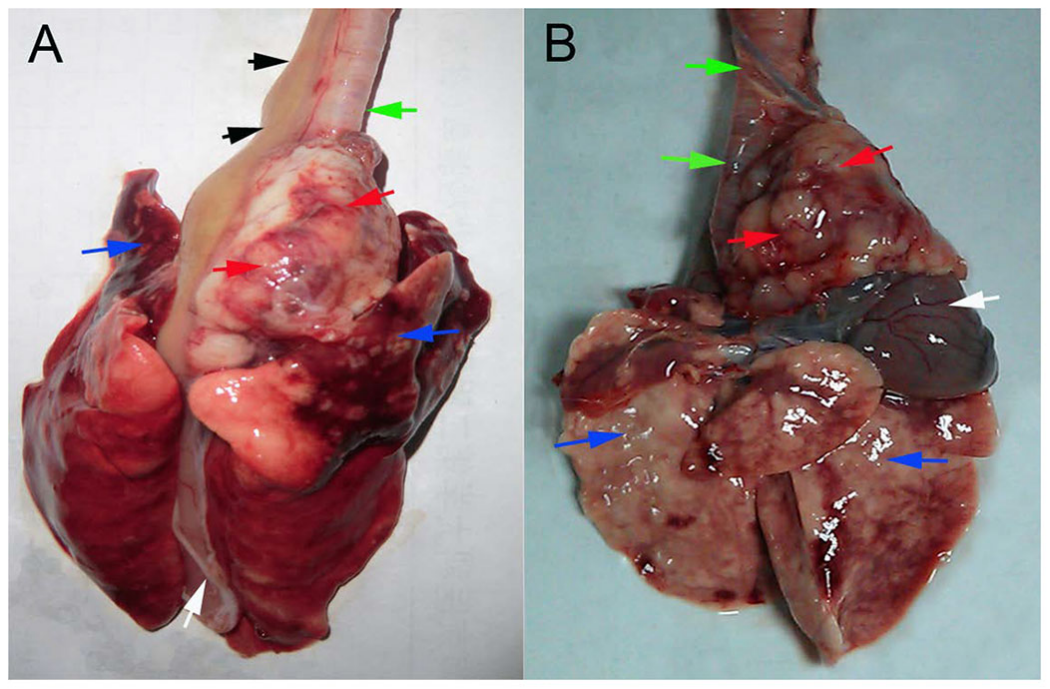
Tumor invasion of adjacent tissues. (A) Invasion was observed into the trachea/bronchus (green arrow) and the pleura and the lung in the upper lobes (blue arrows), but invasion of the heart and the pericardium (white arrow) was not observed. The black arrows point to the esophagus. (B) Tumor invasion was observed into the trachea/bronchus (green arrows) and the heart and the pericardium (white arrow), but invasion of the pleura and the lung (blue arrows) was not observed.

**Figure 6:**
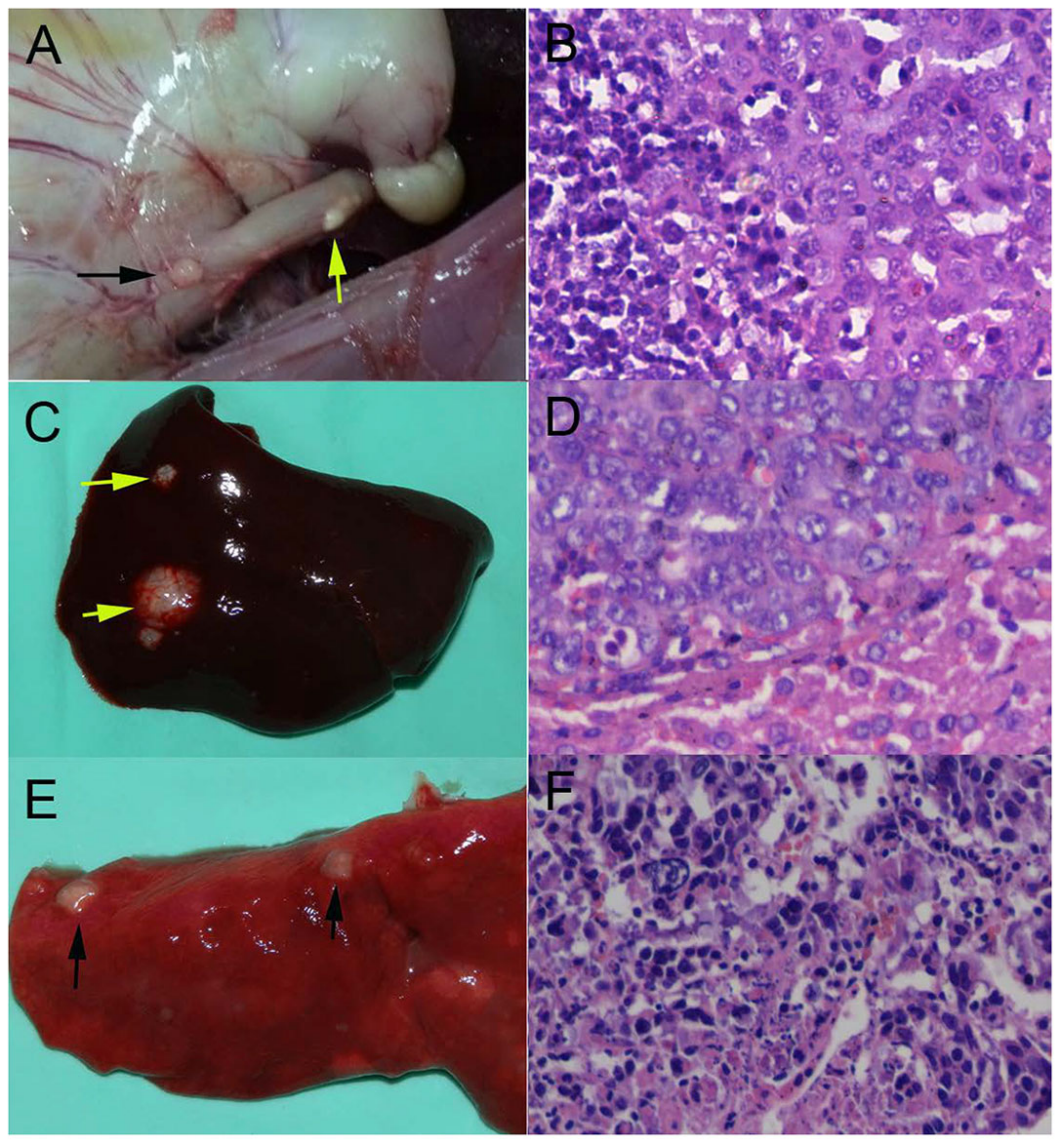
Tumor regional/distant metastases. (A) Macroscopic view of the lymph node metastases (black arrow) and the yellow arrow points to the esophageal tumor. (B) Microscopy observation of lymph node metastases (200x). (C) Macroscopic view of the liver metastases (yellow arrows). (D) Microscopy observation of liver metastases (200x). (E) Macroscopic view of the lung metastases (black arrows). (F) Microscopy view of lung metastases (200x).

The rate of tumor invasion of adjacent tissues (Fig. 5) in the endoscopic group (Table S1) was 53.8% (14/26) and in surgical group (Table S2) was 81.5% (22/27). The difference in the rates between the two groups was 27.7% (95% CI, 3.5% to 51.9%), and the results were statistically significant (*P* = 0.017). The rate of tumor regional/distant metastasis (Fig. 6) in models produced by the endoscopic implantation (Table S1) was 38.5% (10/26) and 51.8% (14/27) by the surgical method (Table S2), and the difference was not significant (*P* = 0.327).

### Survival time of the models

The longest survival time for rabbits that received endoscopic implantation was 81 days and the shortest was 26 days, with a median survival time of 50.0 (95% CI, 46.0 to 54.0) days. For the rabbits that received surgical implantation, the longest survival time was 79 days and the shortest was 21 days, with a median survival time of 46.0 (95% CI, 42.0 to 50.0) days. The difference in the survival times between the two groups was not statistically significant (*P* = 0.34).

## Discussion

The middle third of the esophagus is the most common site for human esophageal squamous carcinoma, so we implanted VX2 tumors in the rabbit thoracic esophagus in this study. Our models are moderate-to-large-sized and were histologically proven to be the squamous cell type. The pathologic features that the models showed such as different degree of esophageal stricture, different tumor growth patterns, with/without invasion of adjacent tissues and regional/distant metastasis, are extremely similar to the clinicopathologic characteristics of human ESC [15]–[17]. The findings indicate that our animal models are reliable for mimicking the disease of human ESC.

There are two types of tumor seeds to select for implantation: VX2 cells or fragments. VX2 cells were generally used for implantation in previous studies [18], [19]. However, it has been reported that the success rate of tumor growth by the implantation of VX2 fragments was higher than by inoculation with intact VX2 cells [20]–[22] in organs such as the liver and the lung. Therefore, we selected VX2 fragments as the tumor seeds for the two implantation methods in this study.

There has not yet been a report on the establishment of an esophageal carcinoma model with VX2 fragments because it is more difficult to implant fragments than whole cells into the wall of the esophagus. The necessary open surgery for the implantation of VX2 fragments is more traumatic than whole cell implantation, and it risks post-surgical complications [23], [24]. As an alternative, the endoscopic method is a minimally invasive procedure but increases the incidence of esophageal perforation when using large needles to implant the VX2 fragments. To avoid severe complications, we introduced a submucosal injection technique for endoscopic implantation [25], [26] to enlarge the space between the mucosal and the muscular layer. When performing implantation with the surgical method, we selected an abdominal operation instead of thoracic surgery, which was accomplished by pulling down the lower thoracic esophagus into the abdominal cavity to decrease the mortality of animal. Although there is no data from other studies that can be used for comparison, the data from the current study indicates that both endoscopic and surgical implantation of VX2 fragments are safe and effective methods for establishment of rabbit esopgageal carcinoma models in the thoracic esophagus.

In current study, the models produced by two methods showed different pathologic features. We believe that the difference was not related to the two implantation procedures, but to the different sites of implantation: the submucosal layer for the endoscopic procedure and the muscular layer implantation for the surgical operation. There are technique limitations to selecting the same site for the two methods: the complication rates of esophageal perforation and mortality increase whether due to puncturing of the muscle layer under endoscopy or the submucosal layer during surgically, in our experience, because the wall of the esophagus is very thin. Our results indicate that implantation site may influence the tumor growth patterns and contribute to the formation of different pathologic characteristics. Thus the preferred model for future studies may depend upon the particular research question being addressed.

The models produced by the endoscopic method showed tumor intra-luminal growth and were generally complicated by severe stenosis, which may allow for research on surgical techniques, endoscopic procedures, stent placement, etc. The models produced by the surgical method showed tumor extra-luminal growth, which was frequently followed by tumor invasion of the adjacent organs and, therefore, can be used for studies on intravascular interventional treatment or palliative surgical procedures. In addition, the rabbits for modeling are animals with normal immunocompetence and their immune responses to therapeutic agents is more predictive of human responsiveness than those of immunodeficient rodent models. Therefore, our models could potentially be used for research on immunotherapy.

In conclusion, endoscopic submuscosal implantation and surgical intramuscular implantation are both safe and effective for establishment of VX2 tumours in the rabbit thoracic esophagus. The models produced by the two methods have different pathologic features, which are similar to that of human ESC, may be attributed to the different tumor implantation sites. We recommend the models for preclinical studies on surgical techniques and minimally invasive treatments.

## Supporting Information

Video S1 **Endoscopic Procedure for VX2 Fragments Implantation.** Using a fine endoscopic needle, 0.5 ml of saline was first injected into the submucosal layer to elevate the mucosa layer. Then, a puncture was made using a large endoscopic needle (a revised needle) across the mucosa into the submucosal layer, and the success of the puncture was confirmed by further elevation of the mucosa when the saline was injected. Next, approximately 0.3 ml of saline containing four tumor pieces was injected into the submucosal layer. Finally, a second 0.3 ml portion of saline was injected to rinse the needles and collect the stuck pieces.
(MP4)

Table S1 **The pathologic characteristics of the models produced by endoscopic method.** For each rabbit, the degree of esophageal stricture, the tumor growth pattern, the status of tumor invasion of adjacent tissues and regional/distant metastasis are given.
(DOC)

Table S2 **The pathologic characteristics of the models produced by surgical method.** For each rabbit, the degree of esophageal stricture, the tumor growth pattern, the status of tumor invasion of adjacent tissues and regional/distant metastasis are given.
(DOC)
