# Supplementary material for: Comparison of Endoscopic Submuscosal Implantation vs. Surgical Intramuscular Implantation of VX2 Fragments for Establishing a Rabbit Esophageal Tumor Model for Mimicking Human Esophageal Squamous Carcinoma
Source: PLoS One. 2014 Jan 24;9(1):e85326. doi: 10.1371/journal.pone.0085326 (PMC3901654; doi:10.1371/journal.pone.0085326)
Supplement: Table S2 — The pathologic characteristics of the models produced by surgical method. For each rabbit, the degree of esophageal stricture, the tumor growth pattern, the status of tumor invasion of adjacent tissues and regional/distant metastasis are given. (DOC) [file pone.0085326.s003.doc]

**Table S2. The pathologic characteristics of the models produced by surgical method**

| **No.** | **Degree of Stenosis** | **Invasion of Adjacent Tissues** | | | | | **Growth Pattern** | **Regional/Distant Metastasis** | | |
| --- | --- | --- | --- | --- | --- | --- | --- | --- | --- | --- |
|  |  | **Pericardium/Heart** | **Trachea/Bronchus** | **Pleura/Lung** | **Diaphragm** | **Vena Cava / Aorta** |  | **Liver** | **Lymph Node** | **Lung** |
| 1 | Mod | N | Y | Y | N | Y | Extra | N | N | N |
| 2 | Mild | Y | Y | N | Y | N | Extra | N | N | N |
| 3 | Severe | N | N | N | N | N | Intra | N | Y | N |
| 4 | Mild | N | Y | Y | Y | N | Extra | N | N | N |
| 5* | Severe | N | N | Y | N | N | Intra | N | N | N |
| 6 | Severe | N | Y | N | N | Y | Extra | N | N | Y |
| 7 | Mod | N | Y | N | N | N | Extra | Y | N | Y |
| 8 | Mild | N | Y | Y | Y | N | Extra | N | N | N |
| 9* | Mod | N | N | N | N | N | Extra | N | N | N |
| 10 | Severe | N | Y | Y | Y | N | Intra | N | N | Y |
| 11 | Mild | N | N | Y | N | N | Extra | Y | Y | N |
| 12 | Mod | N | Y | N | N | N | Extra | N | N | N |
| 13 | Mod | N | N | Y | Y | N | Extra | N | N | N |
| 14 | Mod | Y | Y | Y | N | N | Extra | N | N | Y |
| 15 | Severe | N | N | N | N | N | Intra | N | Y | N |
| 16 | Mod | N | Y | N | N | N | Extra | N | N | N |
| 17* | Mild | N | N | N | N | N | Extra | Y | Y | Y |
| 18 | Severe | N | N | Y | N | N | Intra | N | Y | N |
| 19 | Mod | N | Y | N | Y | N | Extra | N | N | N |
| 20 | Mod | N | Y | N | N | Y | Intra | N | Y | N |
| 21 | Mod | N | Y | N | N | N | Extra | N | N | N |
| 22 | Mild | N | N | Y | N | N | Intra | N | Y | N |
| 23 | Severe | N | Y | N | N | N | Intra | N | N | N |
| 24* | Mod | Y | Y | Y | Y | N | Extra | N | Y | N |
| 25 | Severe | N | N | N | N | N | Intra | N | N | N |
| 26 | Mild | N | Y | N | N | Y | Extra | N | Y | N |
| 27 | Mod | N | N | Y | N | N | Intra | N | Y | N |

Intra = intra-luminal growth; Extra = extra-luminal growth; Mod = moderate; Y = yes; N = no

* indicates animal occuring peritoneal implantation metastasis
